# Using heterogeneous data to identify signatures of dengue outbreaks at fine spatio-temporal scales across Brazil

**DOI:** 10.1371/journal.pntd.0009392

**Published:** 2021-05-21

**Authors:** Lauren A. Castro, Nicholas Generous, Wei Luo, Ana Pastore y Piontti, Kaitlyn Martinez, Marcelo F. C. Gomes, Dave Osthus, Geoffrey Fairchild, Amanda Ziemann, Alessandro Vespignani, Mauricio Santillana, Carrie A. Manore, Sara Y. Del Valle

**Affiliations:** 1 Information Systems and Modeling Group, Analytics, Intelligence and Technology Division, Los Alamos National Laboratory, Los Alamos, New Mexico, United States of America; 2 Center for Nonlinear Studies, Los Alamos National Laboratory, Los Alamos, New Mexico, United States of America; 3 National Security and Defense Program Office, Los Alamos National Laboratory, Los Alamos, New Mexico, United States of America; 4 Computational Health Informatics Program, Boston Children’s Hospital, Boston, Massachusetts, United States of America; 5 Department of Pediatrics, Harvard Medical School, Boston, Massachusetts, United States of America; 6 Geography Department, National University of Singapore, Singapore, Singapore; 7 Laboratory for the Modeling of Biological and Socio-technical Systems, Northeastern University, Boston, Massachusetts, United States of America; 8 Department of Mathematics & Statistics, Colorado School of Mines, Golden, Colorado, United States of America; 9 Núcleo de Métodos Analíticos em Vigilância Epidemiológica Programa de Computação Científica, Fundação Oswaldo Cruz, Rio de Janeiro, RJ, Brazil; 10 Statistical Sciences Group, Computer, Computational, and Statistical Sciences Division, Los Alamos National Laboratory, Los Alamos, New Mexico, United States of America; 11 Space Data Science and Systems Group, Intelligence and Space Research Division, Los Alamos National Laboratory, Los Alamos, New Mexico, United States of America; 12 School of Engineering and Applied Sciences, Harvard University, Cambridge, Massachusetts, United States of America; University of Hong Kong, HONG KONG

## Abstract

Dengue virus remains a significant public health challenge in Brazil, and seasonal preparation efforts are hindered by variable intra- and interseasonal dynamics. Here, we present a framework for characterizing weekly dengue activity at the Brazilian mesoregion level from 2010–2016 as time series properties that are relevant to forecasting efforts, focusing on outbreak shape, seasonal timing, and pairwise correlations in magnitude and onset. In addition, we use a combination of 18 satellite remote sensing imagery, weather, clinical, mobility, and census data streams and regression methods to identify a parsimonious set of covariates that explain each time series property. The models explained 54% of the variation in outbreak shape, 38% of seasonal onset, 34% of pairwise correlation in outbreak timing, and 11% of pairwise correlation in outbreak magnitude. Regions that have experienced longer periods of drought sensitivity, as captured by the “normalized burn ratio,” experienced less intense outbreaks, while regions with regular fluctuations in relative humidity had less regular seasonal outbreaks. Both the pairwise correlations in outbreak timing and outbreak trend between mesoresgions were best predicted by distance. Our analysis also revealed the presence of distinct geographic clusters where dengue properties tend to be spatially correlated. Forecasting models aimed at predicting the dynamics of dengue activity need to identify the most salient variables capable of contributing to accurate predictions. Our findings show that successful models may need to leverage distinct variables in different locations and be catered to a specific task, such as predicting outbreak magnitude or timing characteristics, to be useful. This advocates in favor of “adaptive models” rather than “one-size-fits-all” models. The results of this study can be applied to improving spatial hierarchical or target-focused forecasting models of dengue activity across Brazil.

## Author summary

Dengue virus spreads through mosquitoes in many tropical and subtropical parts of the world, including Brazil. Each year, dengue virus causes seasonal outbreaks that vary in magnitude and timing across the country. This variation makes tailoring preparation efforts for fine spatio-temporal scales challenging. In this study, we described four properties of historical dengue time series at the mesoregion level, the Brazilian subdivision below state, and examined how they varied across the country. We found that the duration and timing of seasonal outbreaks are largely driven by climate factors, while relational properties, i.e., the similarity in outbreak timing and magnitude between two mesoregions, are explained by a mix of mobility patterns and climate similarities. Surprisingly, we found that remote sensing derived products and movement inferred through Twitter were adequate proxies for climate and mobility patterns respectively. Knowledge of how dengue outbreaks differ across the country and the factors that may influence specific outbreak properties may be important for improving efforts to build forecasting and prediction models.

## Introduction

Dengue virus (DENV) is a mosquito-borne virus associated with high morbidity and mortality, and its increasing global burden is of high concern [1]. Transmission in Brazil accounts for 80% of the DENV cases in the Americas, and since 2010 [2] cases caused by all four DENV serotypes have been recorded annually throughout the country. Brazil’s worsening DENV burden has been linked to urbanization and crowding [2, 3], expanding ranges of suitable habitat for DENV-adapted mosquitoes [4], and changing human mobility patterns [5]. These changes have coincided with observed shifts in the epidemiology of DENV in Brazil, including increases in the proportion of infections among younger age groups [6], the proportion of severe cases, and the number of smaller cities reporting transmission [2]. Without effective antiviral treatments and the limited use of an early vaccine, reducing the number of DENV infections depends on effective vector control and public health messaging.

To enhance public health preparedness, there have been several efforts to develop forecasting models of DENV in Brazil [7–10]. Forecasting models integrate single or multiple data streams into statistical or mechanistic frameworks to make short-term (e.g., present to multiple weeks ahead) or long-term (e.g., monthly and year-based) predictions of the number of DENV cases in a given location [11–18]. Data streams aim to capture informative aspects of the transmission process, and for DENV, data streams have included historical DENV case data, socioeconomic indicators, internet search behavior, weather indices, satellite remote sensing data, and entomological observations. Forecasting models can be validated by assessing the accuracy of the predicted versus observed number of cases through time. Another approach is to evaluate how well the model predicts key outbreak targets, such as outbreak onset, peak timing, or duration [19]. Outbreak targets are tied to practical needs, for example, anticipating when peak infections will occur helps establish when maximum resources are needed.

However, it is challenging to develop forecasting models because of the ecological, immunological, and epidemiological complexity of DENV in Brazil. First, Brazil’s heterogeneous climate differentially affects mosquito development and thus DENV dynamics [16, 17]. In locations that experience distinct rainy seasons, particularly the south and southeastern regions of the country, DENV cases exhibit seasonal oscillations, while in tropical regions along the equator, DENV cases are reported year-round. Similarly, climate influences the annual timing of DENV outbreaks. As seen in Southeast Asia [20–22], the onset of annual DENV outbreaks follows a traveling wave throughout Brazil [5, 23]; outbreaks first begin in the northern tropical region, spread counter-clockwise, and end in the more populated coastal states of southeastern and northeastern regions. Yet, DENV transmission itself is highly localized, and this pattern of outbreaks is often the co-occurrence of multiple unrelated outbreaks [24]. Second, although DENV has been recorded in Brazil since 1986, the gradual importation of novel serotypes and the frequent introduction of distinct serotype lineages creates an unpredictable immunological landscape [5, 25, 26], which may contribute to annual differences in timing and magnitude. Finally, forecasting models are typically trained assuming that historical case data are accurate and will be as they become available in real time. This is hardly true in practice, since changes in reporting, misreporting of other co-circulating arboviruses like chikungunya or Zika, and any other hard to capture data vagaries obscure the epidemiological signal [27] in the data. These factors further complicate any forecasting efforts.

This dynamic and irregular landscape of DENV in Brazil suggests that a one-size-fits-all forecasting approach may not offer sufficient flexibility to capture the heterogeneity in historical and future case data. Instead, a suite of goal-oriented forecasting models that each focus on specific practical targets may be beneficial, while simultaneously providing insight into differential drivers of specific outbreak characteristics. For example, the onset of the rainy season may influence the onset of an annual DENV outbreak, but population size or vegetation levels may play a stronger role in explaining the magnitude or the length of the outbreak [28]. Isolating the explanatory power of different drivers on specific outbreak characteristics and identifying which data streams best capture those drivers has the added benefit of increasing the interpretability of their inclusion in any forecasting model. Such a data-driven approach would help narrow the growing space of possible models and data streams while balancing explanatory and predictive power.

Here, we use six years of weekly reported DENV case data in Brazil to identify spatio-temporal structure in DENV dynamics that could help inform the development of goal-oriented forecasting models. First, we analyze the differences in DENV dynamics at the subdivision mesoregion level by exploring four questions that can be tied to measurable properties of the time series. For each mesoregion, we assess how cases are typically distributed across the DENV season (intensity) and what is the regularity in seasonal timing (seasonality). Between pairs of mesoregions, we assess the level of synchronicity in seasonal timing (outbreak timing) and inter-annual changes in the magnitude of cases (outbreak trend). Next, we determine the extent of spatial structure in these properties that may be leveraged in future forecasting models. Finally, we use a regression framework to identify key environmental and human covariates associated with each of these outbreak properties. Together, these results elucidate differential drivers of DENV properties, help identify data sources useful for inclusion in future goal-oriented forecasting models, and provide a template for data-driven analysis of outbreak properties of other infectious diseases.

## Materials and methods

### Data

#### DENV clinical case data

**Fig 1:**
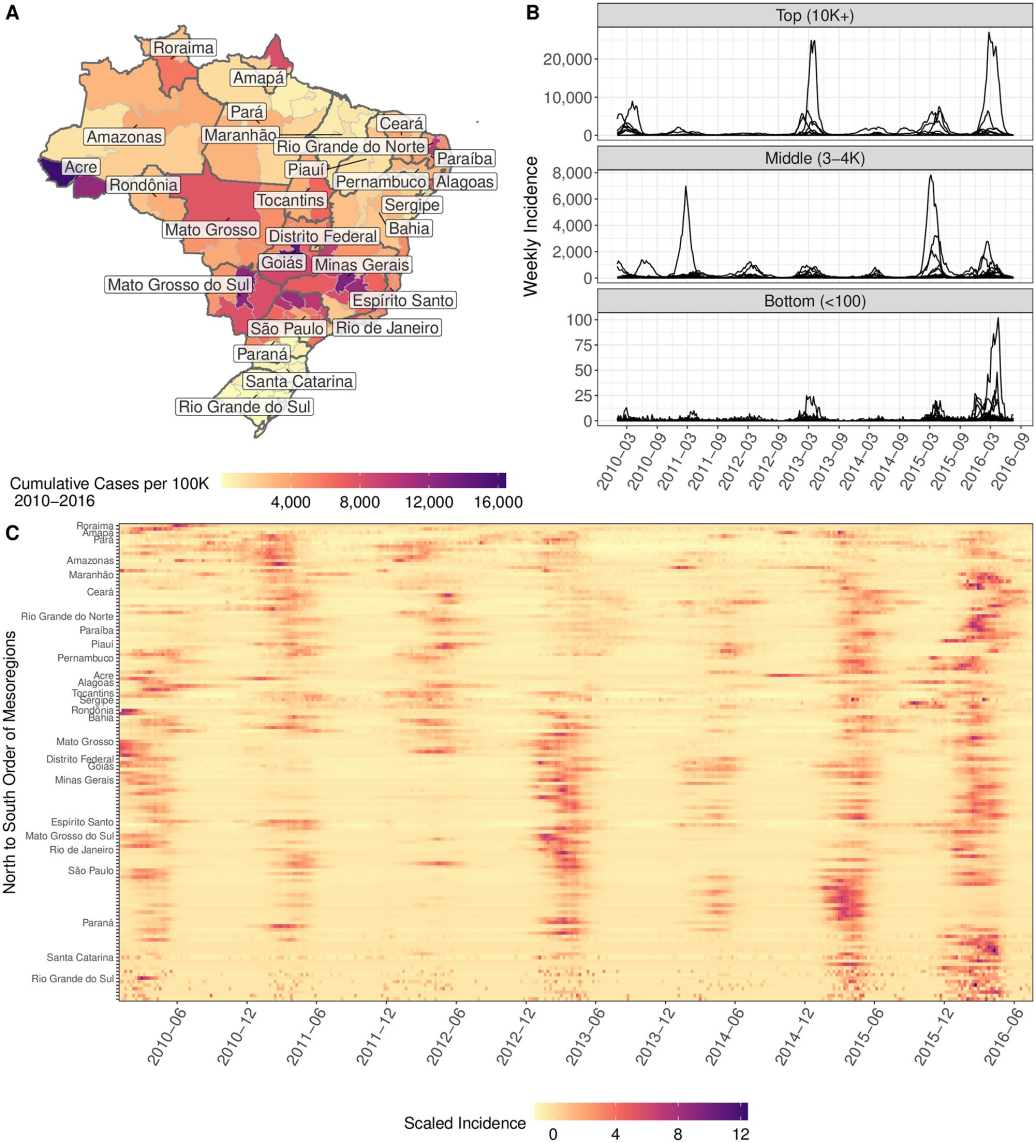
The spatio-temporal heterogeneity of weekly DENV case data across 137 mesoregions from 2010–2016. (A) Cumulative incidence over the six-year study period. States are outlined in darker grey and labeled. The underlying shapefiles with political boundaries are publicly and freely available at Instituto Brasileiro de Geografia e Estatística (IBGE) http://downloads.ibge.gov.br/downloads_geociencias.htm. (B) Examples of mesoregion weekly DENV time series. Mesoregions were sorted according to cumulative incidence over the six-year study period as follows: “Top” mesoregions recorded greater than 10K cases per 100K individuals, “Middle” recorded 3–4K cases per 100K individuals, and “Bottom” recorded fewer than 100 cases per 100K individuals. Top mesoregions come from Acre, Minas Gerais, Rio de Janeiro, São Paulo, Mato Grosso do Sul, and Goiás; Middle come from Rondônia, Amazonas, Ceará, Rio Grande do Norte, Pernambuco, Alagoas, Minas Gerais, São Paulo, and Mato Grosso; Bottom come from Paraná, Santa Catarina, and Rio Grande do Sul. (C) Weekly DENV incidence in each mesoregion. Each time series was transformed to zero mean and unit variance. Mesoregions are ordered from north (top) to south (bottom) by the listed state and then by mesoregion within state.

We obtained the number of weekly confirmed DENV cases at the municipality level (n = 5,570) over the study period of January 2010 to July 2016 from the Brazilian Ministry of Health (Fig 1). We aggregated the case data to weekly time series at the mesoregion level (n = 137), a subdivision below the state level. We focused on the mesoregion level because the majority of DENV transmission in an urban setting has been shown to occur at a localized scale of < 200 m [24], the flight range of *Aedes aegypti*—the primary mosquito vector of DENV in Brazil—is between tens to hundreds of meters [29], and the level of heterogeneity in environmental conditions at higher federative (state) levels may obscure explanatory links between environmental drivers and outbreak properties. To be consistent with the minimum spatial resolution of some of the covariate data, we did not analyze the case data at finer resolutions than mesoregion. Although the mesoregion subdivision was reclassified in 2017 [30], we retained the previous 1990 classification to make our results comparable to earlier work. The spatio-temporal heterogeneity in burden (Fig 1A and 1C) and the presence of seasonal dynamics in some locations (Fig 1B and 1C) motivates the approach of adaptive models rather than a one-size-fits-all.

#### Environmental data

From a previously published environmental data set for Brazil [9, 10], we collected time series of five satellite remote sensing indices (Table 1: green Normalized Difference Water Index (green NDWI), short-wave infrared Normalized Difference Water Index (SWIR NDWI), Normalized Burn Ratio (NBR), Normalized Difference Vegetation Index (NDVI), and % cloudy pixels), and three climatic weather variables (Table 1: daily range in temperature, mean temperature, and relative humidity). Satellite remote sensing data from January 2010 to December 2016 were derived from the multispectral satellites Landsat 5, Landsat 7, Landsat 8, and Sentinel-2, and the source images were accessed via the Descartes Labs Platform [31]. Weather data from April 2009 to April 2017 were retrieved from the National Oceanic Atmospheric Administration’s Global Surface Summary of the Day (GSOD) data set [32]. The remote sensing data came in a temporal resolution of nearly weekly measurements. For the time periods during which satellite data were missing, kriging methods were used to interpolate the known values. From the original spatial measurement resolution of 30 m^2^, each remote sensing index was summarized to obtain the mean weekly value of pixels within a mesoregion. The GSOD data provided daily raw measurements of temperature and humidity from 613 ground weather stations across Brazil. Again, we used kriging methods to spatially and temporally approximate these variables across the country, and then summarized weekly mean values across each mesoregion. Finally, all satellite and weather station variables were combined to give weekly values at the mesoregion level across the six years corresponding to the DENV case data. Full details on how these data were collected, cleaned, and fused can be found in Refs. [9, 10].

**Table 1:** Candidate covariates for explaining outbreak properties. *Measurements* indicate the different ways in which a variable was incorporated as a candidate in the LASSO regression models.

Category	Variable	Description	Measurements
Environmental	Normalized difference vegetation index (NDVI)	Indicator of healthy green vegetation	Mean, Intensity, Seasonality; (Pairwise) Euclidean Distance, Correlation in phase angles, Correlation in trend
	Green normalized difference water index (Green NDWI)	Indicator of water bodies	
	Shortwave infrared normalized difference water index (SWIR NDWI)	Indicator of moisture in vegetation	
	Normalized burn ratio (NBR)	Indicator of burned areas, sensitive to drought	
	% Cloudy pixels	Degree of cloud cover	
	Daily range in temperature	Weekly average of the difference between the maximum and minimum daily temperature in degrees Fahrenheit	
	Mean temperature	Weekly average daily temperature in degrees Fahrenheit	
	Relative humidity	Weekly average relative humidity calculated from the dew point and temperature	
	Human-mosquito contact	Number of mosquitoes per person	
Human—Risk Factors	Total population	Total resident population	Mean
	Rural population	Resident population living in rural areas	
	Population density*	Ratio of the population living in a private home with a density greater than 2 persons per room	
	Garbage collection*	Ratio of private households with waste collection by service provider	
Human—Connectivity	Distance	The great-circle distance between mesoregion centroids (km)	*Pairwise—Median
	Population product	The log product of two mesoregions’ populations	
	Air travel*	The estimated median number of air travelers between two mesoregions	
	Bus travel*	The estimated median number of bus travelers between two mesoregions	
	Twitter activity*	The estimated median number of unique Twitter users between two mesoregions	

These variables indicate aspects of the environmental suitability for mosquito populations, which is a precursor of DENV transmission. Vegetation and water indices provide information on the water dynamics in an area, which can serve as an indicator of suitability for mosquito breeding. Indicators of both healthy and unhealthy vegetation, such as the NBR, can be correlated with urban environments, where mosquitoes not only reproduce, but also indicate areas where human-mosquito contact is likely to occur. Temperature variables may contain information on where the temperature range is suitable for the mosquito life-cycle and virus development.

#### Human-mosquito contact

We estimated the level of human-mosquito contact by approximating the number of *Ae. aegypti* mosquitoes per person following the approach in Zhang et al. [33]. The level of mosquitoes present influences the risk of an infected mosquito biting a human and the risk of an infected human transmitting DENV to a susceptible mosquito. From Kraemer et al. [34], we averaged the 5 km^2^ cell estimates of vector presence (derived from a combination of precipitation, temperature, and an enhanced vegetation index), over each mesoregion. As in Ref. [33], we then assumed that mosquito abundance follows a monthly modulation function depending on the local temperature. The human-mosquito contact variable takes into account the conditions needed for mosquito development, which temperature and NDVI alone are agnostic to. The resulting estimated number of *Ae. aegypti* mosquitoes per person time series covered the same time period as the DENV case data.

#### Demographics

While the presence of mosquito populations are necessary for DENV transmission, human living conditions may modulate the level of contact with mosquitoes, thus influencing DENV risk [35]. To capture socioeconomic effects on DENV transmission, we used publicly available census data from 2010 that provides information on poverty, education, income, and population statistics [36]. We implemented a dimensionality-reduction hierarchical clustering algorithm [37] to map the original 232 variables contained in the census onto four representative variables. This process involved partitioning the 232 variables into 22 groups based on their pairwise similarity and then we further identified four clusters with a set of representative variables with between-cluster correlations less than *r* = 0.6. The four representative variables were Total Population, Rural Population, Population Density, and Garbage Collection. Population density, rural population, and total population are variables that could indicate the level of crowding in urban environments that may facilitate human-mosquito transmission. Areas with low garbage collection service could indicate areas likely to have large amounts of standing water or general substandard housing conditions. Because the census data was provided at the municipality level, we calculated the total population and rural population as the sum over the municipalities within a specific mesoregion, and the remaining two variables as each variable’s mean over the municipalities within that mesoregion.

#### Measurements of human connectivity

Although DENV transmission chains are typically local, high connectivity between two locations could provide frequent seeding of new infections that influence outbreak dynamics in disparate locations. We approximated pairwise connectivity of mesoregions through two different proxy measures: (1) we calculated the great-circle distance [38] in kilometers (km) between two locations’ centroid coordinates and (2) we took the log product of the two locations’ population sizes as inspired by a gravity model that assumes higher flow between larger populations [39].

Additionally, we used empirical measurements of the number of travelers between mesoregions from air travel, bus travel, and Twitter data streams. We did not find a correlation between peak travel months in these data streams and peak cases, and thus used the median number of travelers between locations over the time period for which the data were available. To estimate the average number of air travelers between two mesoregions, we used the monthly number of travelers from January 2014 to December 2016 collected from the Official Airline Guide (OAG) travel database. These data were used to create a network of subpopulations connected by the flux of individuals flying between them, where subpopulations are the combination of multiple municipalities and approximate geographical catchment of the airport [40]. Since the number of monthly travelers between subpopulations *i* and *j* was often not symmetric, we took the median number of monthly travelers from *i* to *j* and from *j* to *i*. To get a median number of travelers between subpopulations over the study period, we calculated the median number traveling on each pairwise route over the 36 months of data. Lastly, to map the monthly number of individuals traveling between subpopulations *i,j* to mesoregions, we assumed that each unique pairwise combination of mesoregions of the municipalities defined by subpopulations *i,j* received the same number of travelers. Patterns of air travelers may be important for determining the role humans play in seeding outbreaks in distant, i.e., inter-state, locations.

To estimate the number of bus travelers between mesoregions, we used a dataset from the National Land Transport Agency of Brazil for January 2013 to September 2016. These data contain the monthly number of passengers per bus route in Brazil [41], where the origin and destination of the route are listed at the municipality level. Passengers are categorized by whether they are full fare, discounted fare, or free fare passengers. For this analysis, we counted every passenger regardless of type. The origin and destination bus stations were mapped to mesoregions. These data were used to generate a network of mesoregions connected by the flux of individuals riding between mesoregions for each month. Since the number of monthly riders between mesoregions *i* and *j* was not symmetrical, we took the median number of monthly riders from *i* to *j* and from *j* to *i*. To get the median number of travelers between the mesoregions over the study period, we calculated the median number riding on each pairwise route over the 45 months of data. Patterns of bus travelers may be important for determining the role humans play in seeding outbreaks in both near and distant locations not serviced by major airports.

We used a geotagged subset of the 1% Twitter feed to estimate the number of travelers via Twitter data between mesoregions. We obtained all geotagged tweets within Brazil from March 2010 to December 2016, which comprised 4.4 million tweets and 1,265,188 unique Twitter users. Each tweet contained the user account id, the tweet content, as well as the latitude and longitude of the account when the tweet was made. From the latitude and longitude, it was possible to infer the mesoregion the user was located in, and by examining multiple tweets of the same id over time, it was possible to infer movement across mesoregions. These data were used to generate a network of subpopulations connected by the flux of individuals moving between mesoregions for each month by examining each change in location for users. As before, we took the median number of monthly travelers from meosoregion *i* to mesoregion *j* and from mesoregion *j* to mesoregion *i*. To get the median number of travelers between the mesoregions over the study period, we calculated the median number traveling on each pairwise route over the 88 months of data. While impossible to determine whether an individual moves by car, bus, or air, movements discerned through Twitter provide a view of mobility not constrained by a specific transportation mode.

### Characterizing spatio-temporal properties of DENV dynamics

#### Outbreak intensity

**Fig 2:**
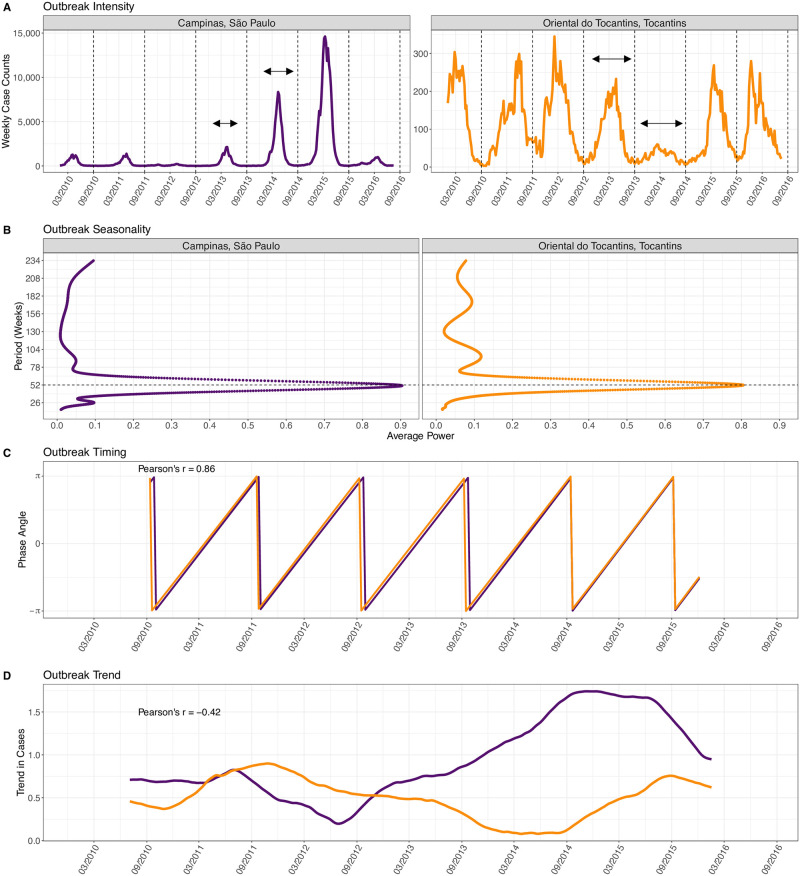
Outbreak properties of DENV case time series for mesoregions Campinas (São Paulo, orange) and Oriental do Tocantins (Tocantins, purple). (A) *Outbreak intensity*, conceptually represented by the arrows, quantifies the distribution of cases over the DENV season, marked by the dashed lines. Oriental do Tocantins has a lower intensity (i.e., more equal distribution of cases) than Campinas. (B) *Outbreak seasonality* quantifies how well the seasonal time series fits a 52-week period. Campinas average power at the 52-week period (0.89) is higher than Oriental do Tocantins (0.81), representing a more predictable seasonal pattern. (C) The phase angles of the two mesoregions’ time series, measuring *outbreak timing*, are in high correlation (*r* = 0.86) with each other. The average phase difference between the two indicates that Campinas is on average 1.2 weeks behind Oriental do Tocantins. (D) The trend components, measuring *outbreak trend*, of the two mesoregions are negatively correlated over the six years of study. The time series are scaled prior to extracting the trend component.

Outbreak intensity quantifies how DENV cases are distributed throughout the year. Mesoregions with high outbreak intensities have shorter outbreaks with steeper rises to the peak, while mesoregions with low outbreak intensities have cases more evenly distributed throughout the year (Fig 2A). We first calculated an outbreak intensity metric [28] for each DENV year between September 1-August 31. For DENV year *j*, we calculated the proportion of yearly cases in mesoregion *m* that occurred in each week *i* as *p*_*ij*_. The outbreak intensity of year *j*, *v*_*j*_, was calculated as the inverse of the Shannon entropy *v*_*j*_ = (∑_*i*_ *p*_*ij*_ log *p*_*ij*_)^−1^. Thus, outbreaks with high intensity correspond to low entropy values and outbreaks with low intensity correspond to high entropy values. We normalized *v*_*j*_ to be between 0 and 1 across all mesoregions, with 1 and 0 representing the mesoregions with the maximum and minimum intensity values across the study period respectively. We calculated the outbreak intensity metric by averaging the normalized intensity over the six-year study period. Mesoregions with years in which low case numbers cluster into a few weeks can return highly-skewed entropy distributions, thus we did not calculate an outbreak intensity value for a mesoregion in a year in which there were fewer than 150 total DENV cases (n = 139). With this criteria, eight mesoregions did not exceed 150 DENV cases in any year over the study period and thus did not have an overall mean outbreak intensity value. We tested other thresholds ranging from 5–200 total DENV cases, and found the distribution of mean outbreak intensity values was not sensitive to the specific criterion between the range of 50–200 total cases (S11 Fig).

#### Outbreak seasonality

Outbreak seasonality quantifies how strongly a mesoregion’s time series adheres to a 52-week cycle [42]. Seasonal oscillations in mesoregions with strong outbreak seasonality occur at regular intervals, while seasonal oscillations in mesoregions with weak seasonality do not. To calculate a seasonality metric, we used the R WaveletComp package [43] to conduct a wavelet analysis of the frequency structure of the time series. We reduced each mesoregion’s standardized time series into its periodic signal for periods of 26 to 234 weeks using the Morlet wavelet function. We took the seasonality metric as the average power value at the 52-week period (Fig 2B).

#### Outbreak timing

Outbreak timing quantifies the level of synchronicity in seasonal timing between two mesoregions’ time series. Specifically, it measures the correlation between the time series’ frequency components (phase angles [43], Fig 2C). From each mesoregion, we decomposed the DENV case time series into the phase angles at the 52-week period. We then calculated the Pearson’s correlation value of the phase angles for all pairwise combinations of mesoregions (n = 9,316). In addition, we computed the average phase difference between pairs of mesoregion phase angles. A positive phase difference means that the reference mesoregion on average leads the other mesoregion in terms of its seasonal DENV pattern, which may or may not be epidemiologically linked. We found there was a strong negative correlation between the absolute pairwise correlation of phase angles and the average phase difference (r = −0.946, p < 2.2*e*^−16^), and therefore either metric could be used to assess outbreak timing.

#### Outbreak trend

Outbreak trend quantifies the level of synchronicity in increases or decreases of outbreak amplitudes between two mesoregions’ time series. When the seasonal pattern is removed from a time series, the remaining trend component captures the directional magnitude changes (Fig 2D). For each mesoregion, we scaled the time series so that increases or decreases in DENV case counts would be on the same scale and then extracted the trend component using the decompose additive function in the R Stats package [44], assuming a seasonal cycle of 52 weeks. We calculated the Pearson’s correlation value of outbreak trends across all pairwise combinations of mesoregions.

### Determining spatial structure of DENV properties

We analyzed the spatial structure of the outbreak properties using spatial autocorrelation and clustering analyses. First, we used the spatial correlation Moran’s *I* [45] to test the spatial randomness of each of the four properties. For each mesoregion, we summarized singular values of outbreak timing and outbreak trend by taking the average over all its pairwise correlations for each property. We used the mean outbreak intensity and mean outbreak seasonality as described above. Second, we considered all four properties together and used a k-means clustering algorithm with 25 random starts to partition the mesoregions into clusters of similar overall outbreak profile. We chose the number of clusters to analyze based on groupings that had a high measure of compactness, defined as the ratio of the sum of squared distances between clusters over the total sum of squared distances, and validated the groupings using the silhouette coefficient [46]. A silhouette coefficient > 0 indicates that an observation is well clustered, with 1 being the highest value; a silhouette coefficient < 0 indicates the observation is in the wrong cluster.

### Identifying covariates of DENV outbreak properties

For each of the four outbreak properties, we developed a series of statistical models to help determine how explainable a property was by environmental and human drivers. To assess the relative contribution of different categories of covariates, we built models using subsets of environmental, human-risk, and human-connectivity variables (Table 1).

To transform each of the environmental variable time series into summary statistics to be used in the models, we used the mean of the six-year time series and computed a number of additional summary statistics that mirrored the outbreak properties (Table 1). For each environmental variable and mesoregion, we calculated its intensity and seasonality. For each environmental variable and pair of mesoregions, we calculated the pairwise correlations between phase angles and the pairwise correlations between trends, as well as the Euclidean distance between the raw values as a measure of overall similarity. All property calculations used the same methodology as described for the DENV case time series. In total, we computed 54 environmental candidate covariates.

Because of the large candidate covariate space, we used LASSO regression to minimize model complexity. We followed the same procedure for each property analysis. First, we normalized all candidate covariate data to be between 0 and 1. We split the data into an 80% training and 20% testing data set randomly by mesoregion and fit a LASSO regression model using five-fold cross validation on the training data. We then tested the model using the one-standard deviation lambda value from the training set on the held-out test set and captured two error statistics: the out-of-sample *r*^2^ and the relative mean squared error (MSE). Because the random fold selection returned different values of lambda and covariates with nonzero coefficients, we repeated this model-fitting procedure 100 times to calculate the proportion of times that a variable was included in the final model. All LASSO models were fit using the glmnet R package [47].

## Results

### Outbreak properties show variation across mesoregions

**Fig 3:**
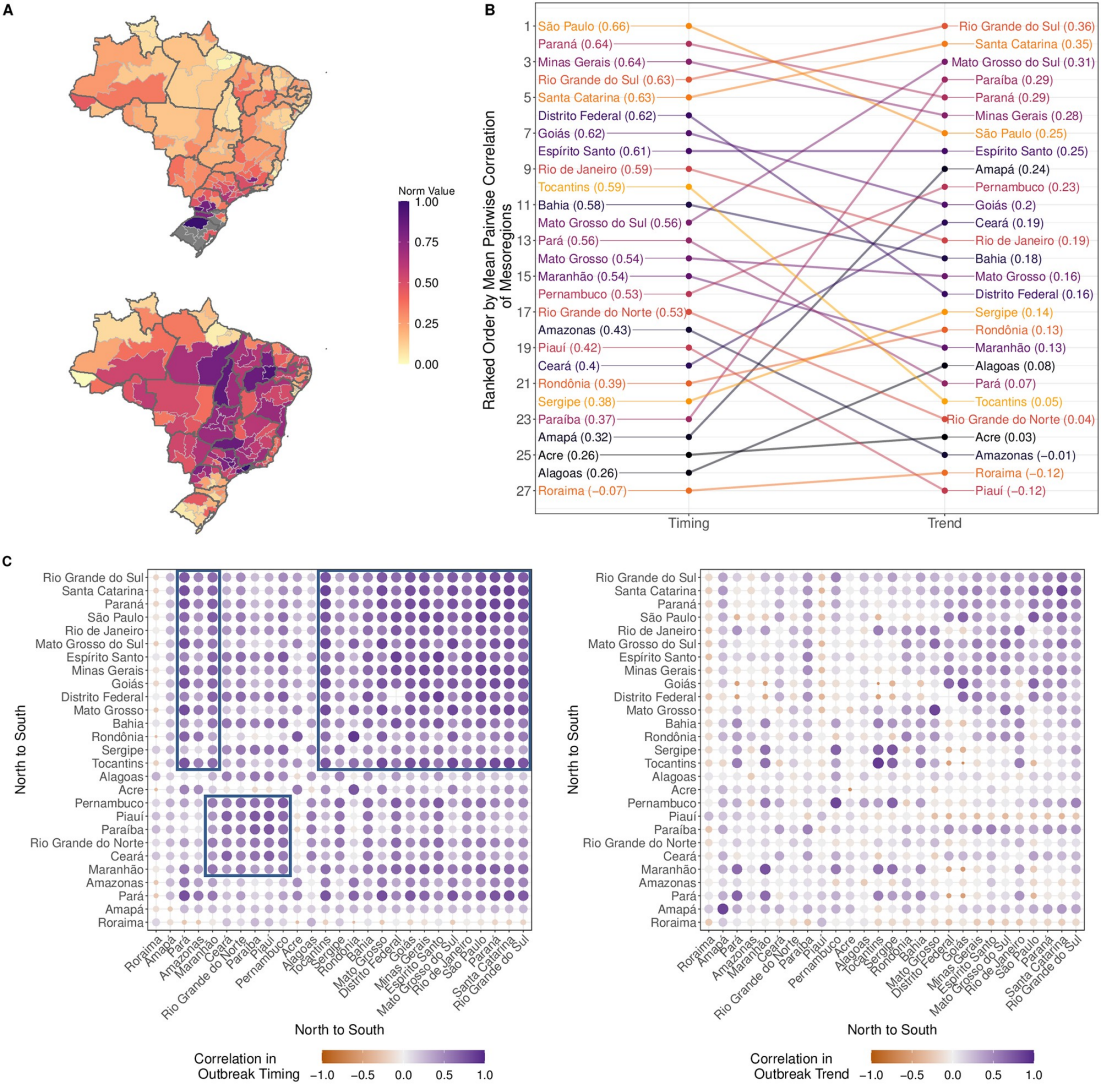
Mesoregion DENV outbreak properties. (A) Normalized mesoregion outbreak intensity (top) and outbreak seasonality values (bottom). Dark grey lines designate state boundaries. Medium grey areas indicate mesoregions where fewer than 150 annual cases were reported each year of the study period. The underlying shapefiles with political boundaries are publicly and freely available at Instituto Brasileiro de Geografia e Estatística (IBGE) http://downloads.ibge.gov.br/downloads_geociencias.htm. (B) State ranking of outbreak timing and outbreak trend by average mesoregion correlations. Colors only serve to visually connect a state’s ranking in outbreak timing to its ranking in outbreak trend. The mean correlation estimate is included in the parenthesis next to each state label. (C) Heatmap of pairwise correlations between outbreak timing (left) and outbreak trend (right). For each pair of states, the mean correlation is calculated by averaging over all combinations of mesoregion comparisons between the two states. Three possible clusters of high correlation in outbreak timing are indicated by black boxes.

From January 2010 to July 2016, we found distinct patterns of outbreak intensity and seasonality at the mesoregion level (Fig 3A). Intensity roughly followed a latitudinal gradient with higher intensity values in the southern and southeastern parts of the country. Outbreak seasonality was stronger in the southeastern and northeastern parts of Brazil, while weaker in the northern and southern extremes of the country where DENV tends to be less seasonal. The minimum seasonality power value was 0.05 in Vale do Juruá, Acre; the maximum seasonality power value was 1.13 in Vale do Paraí ba Paulista, São Paolo. The combination of low intensity and low seasonality in the northern tropic region of Brazil is consistent with the region’s low level of year-around DENV transmission.

Patterns in pairwise correlations of outbreak timing and outbreak trend also varied across Brazil (Fig 3B). For both outbreak properties, the correlations were significantly higher for intra-state comparisons (n = 959) versus inter-state comparisons (n = 17,810) (Both tests: Wilcoxon rank sum test, p < 2.2*e*^−16^). Consistent with the similarity between the pairwise phase angle correlation (outbreak timing) and the average phase difference (temporal lag), mesoregions showed both higher outbreak timing correlations and smaller temporal lags (r = 0.54, p < 2.2*e*^−16^) as a function of decreasing distance.

To visualize the geographic patterns of mean correlations in outbreak timing and outbreak trend, we calculated state-level mean correlation values by averaging over all pairwise mesoregion comparisons of those mesoregions comprising a state. The relative ranking of states was not consistent between outbreak properties (Fig 3B), although four of the top five most correlated states in outbreak timing were also in the top five most correlated states in outbreak trend. Neither the relative ranking of outbreak timing nor outbreak trend followed a clear geographic gradient. Instead, high correlations occurred between smaller clusters of mesoregions spanning several states (Fig 3C). We noted three main outbreak timing clusters: (1) mesoregions which are located in the southern and southeastern parts of the country, including Rio de Janeiro and São Paulo; (2) a group of mesoregions within three northern states that are also in high synchrony with group (1); and (3) a group of mesoregions in six northeastern states. We did not find the same level of clustering in outbreak trend. However, correlations in outbreak trend tended to be higher between neighboring mesoregions, as shown by higher correlations of intra-state outbreak trends (diagonal).

### Towards incorporating spatio-temporal structure into prediction models

As shown in Fig 3, outbreak properties have distinct spatial patterns. We used the Moran’s *I* with a binary adjacency weighting scheme to assess the level of spatial autocorrelation for each property. All properties showed strong evidence of positive spatial clustering, i.e., high values near high values and low values near low values (Table 2). We found similar results when assigning weights based on distance between mesoregion centroids.

**Table 2:** Evidence of spatial structure by outbreak property. Moran’s *I* can be between -1 and 1. A positive Moran’s *I* indicates values are more spatially clustered than would be expected if underlying processes were random; a negative value indicates values are more spatially dispersed.

Property	Moran’s *I*	P-Value
Intensity	0.69	*p* < 2.2*e*^−16^
Seasonality	0.50	*p* < 2.2*e*^−16^
Timing	0.36	*p* = 1.8*e*^−12^
Trend	0.44	*p* < 2.2*e*^−16^

**Fig 4:**
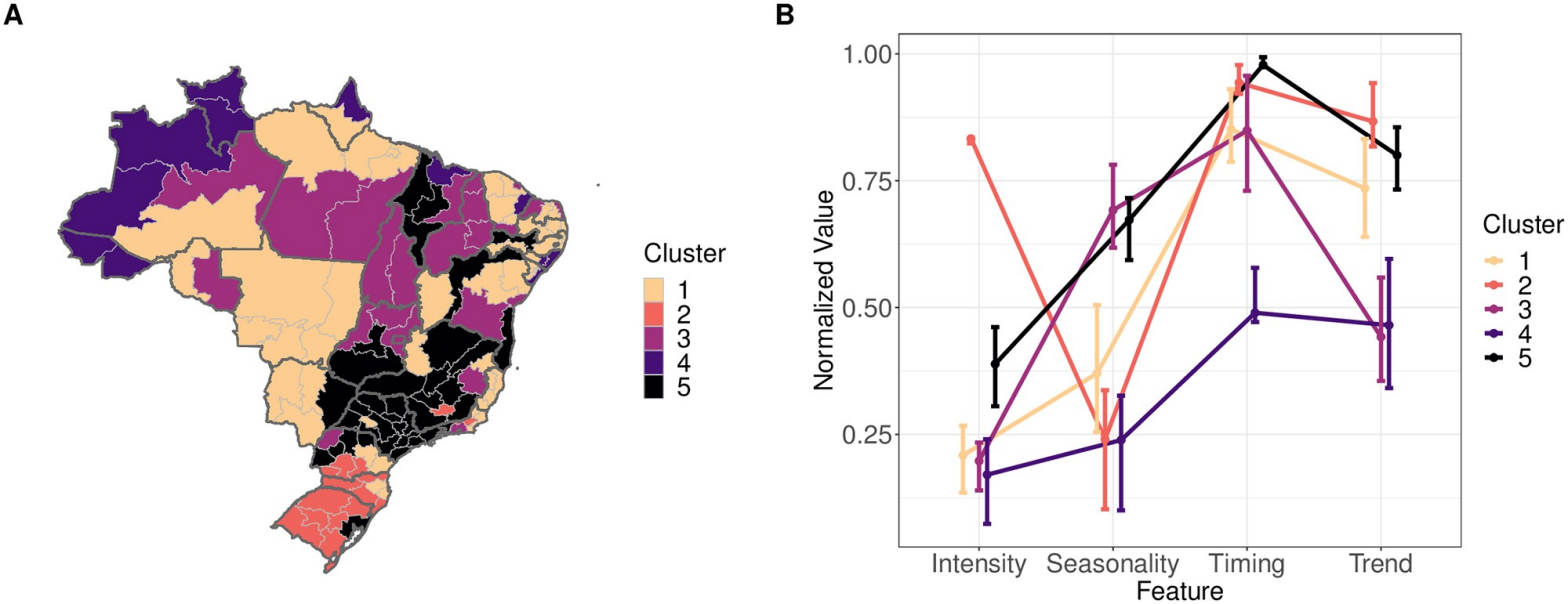
Mesoregion clusters based on outbreak properties. (A) Clusters of mesoregions with overall similar epidemic properties. For mesoregions in the southernmost portion of Brazil where intensity values were not calculated (Fig 3A), intensity measures were imputed for the purpose of clustering as the mean of the top 5% of intensity values over all mesoregions. The underlying shapefiles with political boundaries are publicly and freely available at Instituto Brasileiro de Geografia e Estatística (IBGE) http://downloads.ibge.gov.br/downloads_geociencias.htm. (B) Property values were normalized between 0 and 1. Dots represent the mean and error bars represent the inter-quartile range of each property by cluster.

We used k-means clustering to identify groups of mesoregions that shared similar outbreak properties. There was modest support for five groups of mesoregions based on overall outbreak similarity. With k = 5 clusters, the compactness score, a measure of differences between clusters was 66% and the average silhouette width, a measure of how well-grouped the observations are, was 0.31. Five mesoregions showed evidence of grouping with another cluster: Sertão Paraibano (Paraíba—Northeast), Noroeste de Minas (Minas Gerias, Southeast), Noroeste Paranaense (Paraná—South), Parntanais Sul-Mato Grossense (Mato Grosso de Sul—Central West) and the Distrito Federal (Central West). These mesoregions often were on the fringes of their grouping (Fig 4A). The southernmost cluster (cluster 2) is characterized by high intensities, a weak seasonal signal, and higher correlations in outbreak timing and trend (Fig 4B). Cluster 4, which contains Amazonian mesoregions that border other countries, showed the most dissimilarity overall. The outbreak dynamics of these mesoregions may be affected by importations from neighboring countries [5].

### Models of outbreak properties

#### Explanatory power of outbreak properties varies by covariate set

For each outbreak property, we built a suite of models to determine the relative importance of different categories of covariates and their overall predictive ability (Table 3). Considering the environmental variables, the variation in outbreak intensity was marginally more explained by the intensity of the environmental variables (model 1: 54.4%) than by the annual averages of the environmental variables (model 3: 53.0%). However, the variation in outbreak seasonality was better explained by the annual averages of the environmental variables (model 2: 33.2%) rather than by the seasonality of the environmental variables (model 3: 24.1%). These improvements in the relative MSE were significant based on Paired Wilcoxon signed rank tests (intensity: p < 0.001, seasonality: p < 0.001). Human risk factors on their own or in conjunction with the environmental intensity variables did not further improve the fit for the outbreak intensity model (MSE Paired Wilcoxon signed rank test, p < 0.001), but combined with the environmental annual averages, improved the outbreak seasonality model (MSE Paired Wilcoxon signed rank test, p < 0.001).

**Table 3:** Performance of outbreak property LASSO models. Models are listed in descending order of the mean out-of-sample correlation. The ranked order between the out-of-sample *r*^2^ (higher indicates a better fit) and the relative MSE (lower indicates a better fit) is not exact. Standard deviation of the 100 model fits are listed in parentheses.

Property		Covariates	Relative MSE	Out-of-sample *r*^2^
Intensity	1	Environmental: intensity	0.024 (0.008)	0.544 (0.122)
	2	Environmental: intensity & human-risk factors	0.025 (0.008)	0.543 (0.123)
	3	Environmental: annual average	0.024 (0.008)	0.530 (0.117)
	4	Human-risk factors	0.045 (0.015)	0.137 (0.086)
Seasonality	1	Environmental: annual average & human-risk factors	0.079 (0.018)	0.378 (0.134)
	2	Environmental: annual average	0.084 (0.018)	0.332 (0.134)
	3	Environmental: seasonality	0.094 (0.019)	0.241 (0.126)
	4	Human-risk factors	0.112 (0.016)	0.073 (0.096)
Timing	1	Correlation between environmental phase angles & human-connectivity factors	0.100 (0.004)	0.340 (0.017)
	2	Human-connectivity factors	0.108 (0.004)	0.297 (0.017)
	3	Correlation between environmental phase angles	0.114 (0.004)	0.250 (0.016)
	4	Euclidean distance between environmental time series	0.119 (0.004)	0.214 (0.014)
Trend	1	Correlation between environmental trend & human-connectivity factors	0.764 (0.019)	0.107 (0.013)
	2	Correlation between environmental trend	0.773 (0.019)	0.096 (0.013)
	3	Euclidean distance between environmental time series	0.777 (0.016)	0.089 (0.012)
	4	Human-connectivity factors	0.787 (0.017)	0.081(0.011)

We conducted a similar analysis of the inter-mesoregion properties using subsets of covariates. We first tested whether similarities in raw measurements of the environmental variables, as measured by the Euclidean distance between the environmental time series, or similarities in the properties of environmental variables better predicted the outbreak timing and outbreak trend correlations between two mesoregions. In both cases, similarity in the decomposed time series component (timing model 3, trend model 2) was significantly more informative of the property correlation (Paired Wilcoxon signed rank tests, p <2.2*e*^−16^). Human factors indicative of connectivity between mesoregions explained more of the variation in outbreak timing (model 2) than the environmental variables alone. Finally, the combination of correlations between the decomposed environmental time series and the human connectivity covariates further improved the fit, raising the out-of-sample *r*^2^ to 0.34 for outbreak timing. Although the addition of the human-connectivity factors to correlations between the environmental trend component improved the out-of-sample *r*^2^ correlation for outbreak trend to 0.107 (trend model 1, MSE Paired Wilcoxon signed rank test, p <2.2*e*^−16^), these absolute gains were minimal compared to the other outbreak properties.

#### Determinants of outbreak properties

Because outbreak properties capture different components of the DENV case time series, we hypothesized that the covariates would differ between the outbreak property models. We analyzed the inclusion of covariates from the model which best explained each outbreak property as listed in Table 3.

#### Outbreak intensity

**Fig 5:**
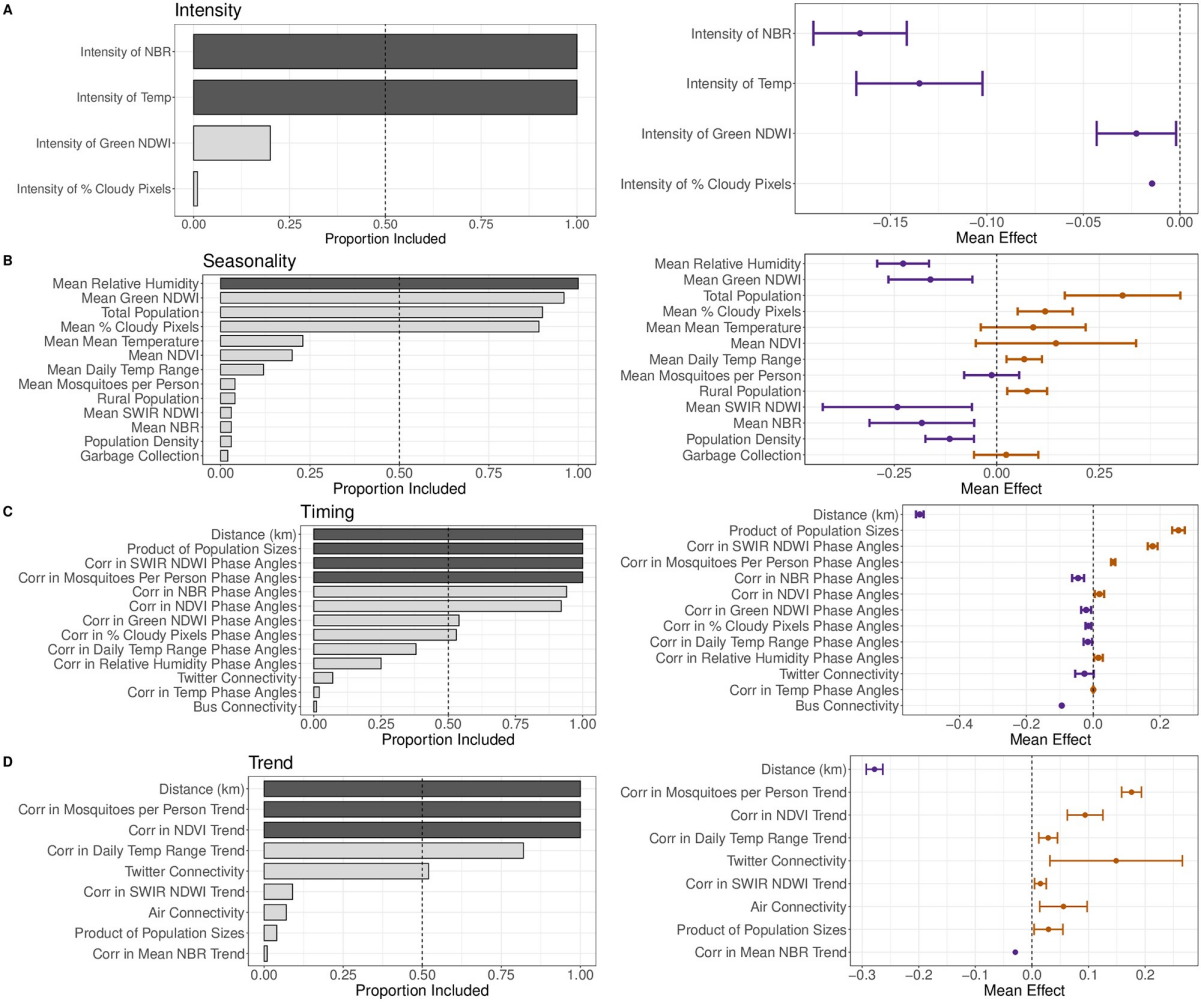
Coefficients of the best-performing outbreak property models. (**Left**) Covariates are ordered by the proportion of times they were included in the final model. Dark bars indicate a covariate was included in all 100 of the LASSO fits. (**Right**) The mean and standard deviation coefficient when the covariate was included in the model. Orange bars indicate a positive effect on the outbreak property value; purple bars indicate a negative effect on the outbreak property value.

The top two predictors of outbreak intensity were the mean temperature and the intensity of the mean normalized burn ratio, an indicator of drought (Fig 5A). Both variables were included in 100% of the fitted models and had negative effects on outbreak intensity, contributing a predicted decrease in the intensity of DENV outbreaks for increases in the intensity of these environmental features. The intensities of the green NDWI and the percent of cloudy pixels variables were also included, however, less than 25% of the time.

#### Outbreak seasonality

Models that predicted outbreak seasonality occasionally consisted of a subset of all nine environmental summary variables and four human-risk factors (Fig 5B). Environmental variables associated with the presence of rain, such as the relative humidity, green NDWI, and average percent of cloudy pixels, were included most often in the models, 100%, 96%, and 89% of the time respectively. The total population of a mesoregion was the most included human-risk factor variable, present 90% of the time. Increases in average relative humidity and green NDWI decreased the strength of DENV case seasonality in a mesoregion, while increases in the average percent of cloudy pixels and the total population size increased the strength of DENV case seasonality. The annual averages of green NDWI, NDVI, and the daily temperature range are highly correlated with each other (*r* = 0.75–0.94, S1 Fig), but in all but two of the 100 LASSO fits, at least one of the coefficients was set to zero. This suggests that these three parameters are being treated as a group by LASSO. Mean temperature and the human-mosquito contact variables are also correlated with each other (*r* = 0.79, S1 Fig), but only appear with nonzero coefficients together three times out of the 100 LASSO fits.

#### Outbreak timing

The top four predictors of outbreak timing were included in 100% of the models (Fig 5C). Distance had a negative effect on outbreak timing correlation, while the product of the population sizes had a positive effect. Correlation of SWIR NDWI and estimated mosquitoes per person phase angles also had positive effects on correlation of outbreak timing. Of the remaining variables included in more than 50% of the model fits, the other four were environmental.

#### Outbreak trend

Five variables were included in the outbreak trend model more than 50% of the time (Fig 5D). Distance again had the strongest negative effect on the values of outbreak trend, while correlations of trends of the two environmental variables, NDVI and the number of mosquitoes per person, had positive effects. Connectivity estimated using movement inferred through Twitter had a stronger mean effect than the trend correlations in NDVI and daily temperature range, although with a wide standard deviation.

## Discussion

We reduced DENV case time series to outbreak properties to identify and analyze targets that can be practically incorporated into future goal-oriented DENV forecasting models. Overall, we found that outbreak shape (intensity) and regularity in seasonal oscillations (seasonality) vary across the country independently of each other. Similarly, mesoregions that have a high degree of synchronicity in their seasonal oscillations (outbreak timing) do not demonstrate the same degree of synchronicity in inter-annual magnitude changes (outbreak trend). In addition, our analysis revealed spatial structure of individual outbreak properties and of an overall outbreak profile that could be leveraged in hierarchical modeling approaches. Finally, we determined that environmental and human associated variables have different roles in shaping these outbreak properties. Intra-mesoregion properties, i.e., outbreak intensity and seasonality, were best explained by satellite remote sensing and climatic variables. Inter-mesoregion properties, i.e., outbreak timing and trend, were best explained by a combination of environmental variables and human-connectivity proxies such as the distance between locations and the product of the population sizes. Altogether, this analysis suggests that developing forecasting models around individual targets may be more effective for single or groups of mesoregions based on regional needs.

Our results revealed that there is wide variation in the predictability of outbreak properties. Outbreak intensity was the most explainable property. In contrast with influenza, we did not find evidence that population size or density played a strong role [28] in predicting outbreak intensity. Theory has traditionally assumed that vector-borne diseases are transmitted in a frequency-dependent manner, where the number of human contacts with mosquitoes are independent of the population size, while person-to-person transmitted diseases like influenza are assumed to be transmitted in a density-dependent manner [48–50]. However, for DENV, whose vector *Ae. aegypti* thrives in urban environments [51], these assumptions may not hold across the spatial heterogeneity of urban landscapes. Romeo-Aznar et al. found evidence of dependencies on the force of infection between human density and mosquito abundance at fine urban scales in Delhi, with differences stemming from socio-economic conditions [52]. One possibility therefore is that we analyzed outbreak intensity at a higher spatial resolution, where differing relationships between density and outbreak dynamics obscurred any signal. Further, population density may not have been a significant covariate because its operational definition used by the Instituto Brasileiro de Geografia e Estatística [36]—the ratio of the population living in a private home with a density greater than 2 persons per room—is more specific than a standard population per geographic unit area definition. When looking at the spatial variation of population density (S9 Fig), high values correspond to areas in the north of the country, with medium values in urban areas such as Rio de Janeiro and São Paulo. Total population size, however, was an important factor in predicting the seasonality of outbreaks, consistent with Ref. [3] that found epidemic persistence is associated with population size. The higher populations of urban environments may lead to consistent annual outbreaks because of widespread water retention storage and the presence of substandard housing or sewerage and management systems. Additionally, in urban environments, subsequent annual outbreaks may appear at the mesoregion level, although occurring in different pockets throughout the city as immunity is gained [24, 35].

We found NBR to predict outbreak intensity and outbreak timing. Locations with lower values of NBR intensity, indicating more consistent NBR measurements, had higher intensity DENV outbreaks. This may be because NBR, as an indicator of land cover with less vegetation, could reflect places where ambient temperatures are warmer due to the urban island effect, which may promote mosquito populations that help fuel larger outbreaks [3]. Similarly, we found the intensity of temperature to be negatively associated with concentrated outbreaks. Mesoregions with changing temperatures may indicate areas where temperature is seasonally inconducive for breeding, survival, and transmission [53–55]. Additionally, NBR, as an indicator of drought, could indicate areas where storage of rainfall in containers may facilitate mosquito breeding [56]. Monitoring for anomalies in NBR within cities could inform whether a DENV season is likely to be more or less intense than usual, which has implications for healthcare surge capacity.

Our findings on outbreak timing across Brazil agree with prior studies that show the presence of a traveling wave and a decaying relationship in outbreak timing with distance [23, 57]. Similarly, we found that environmental variables related to precipitation, such as SWIR NDWI, vector presence, and markers of human connectivity were needed to explain the variation in outbreak timing. At the mesoregion spatial scale, Churakov et al. [23] found that precipitation played a stronger role than human mobility. However, in our analysis, the effects of distance and the product of the population sizes were 2.9 and 1.4 times bigger respectively than the correlation in SWIR NDWI, which is not a direct measure of precipitation but captures the moisture content in vegetation. The inclusion of multiple indirect precipitation satellite remote sensing metrics, including NDVI and NBR, may have diluted the effect of any single one, but suggest that each are capturing subtle water-related differences important to the DENV transmission cycle. SWIR NDWI may be capturing local similarities in precipitation and humidity. Rainfall impacts the life-cycle of *Ae. aegypti* in the egg, larva, and pupa stages and has been shown to precede the occurrence of DENV cases in parts of Brazil [58, 59].

Anticipating long-term changes in magnitude is an important target for a forecasting system, yet correlation in outbreak trend was the least explainable property. We introduced a new metric for measuring synchrony in trend compared to prior studies which have measured synchrony as the correlation in the raw time series. We chose a different definition to ensure that spurious relationships were not inflated by similarities in the seasonal pattern. In our models, environmental and human connectivity variables were able to explain 11% of the variation in outbreak trend, which was slightly less than the models correlating the time series. Interestingly, connectivity estimated through Twitter activity was included in over 50% of the models. Compared to the connectivity estimated through bus and airline proxies, the Twitter connectivity matrix is more complete and may be a better indicator of individual movement as it is not as biased towards mesoregions with transportation hubs. However, the large amount of unexplained variation suggests that key additional data streams are absent. Given the complex interaction between human susceptibility and circulating DENV serotypes, data streams that capture serological information and current circulating pathogen genotypes could improve the ability to explain trend. This type of data is difficult to receive in real time over a broad geographic scale, and may be more suited for modeling at a finer spatial resolution. Additionally, reporting differences across mesoregions and years could obscure the true signal.

This study had several limitations. First, our analysis was only based on six years of data for which we had overlapping clinical, satellite remote sensing, weather, and human connectivity data, potentially missing multi-year DENV cycles that have been shown to occur in other regions and climatic weather events such as El Niño [21, 22, 60, 61]. Second, to increase the parsimony and interpretability of the models, we only considered the weekly mean of the environmental time series. However, there are environmental limits that govern DENV dynamics, such as where temperature is not conducive to mosquito survival [54]. Statistics that capture the upper and lower bounds of environmental conditions may be more informative variables. Third, we assumed a linear relationship between covariates and DENV properties. As mentioned above, there may be hard cutoffs where a linear relationship does not exist. We explored fitting polynomial LASSO models to include interactions between variables, but found the increase in model performance to be negligible. Finally, our study is limited by the accuracy of the DENV surveillance data. Because all reported DENV cases may not be confirmed via laboratory testing, the DENV cases may be more reflective of general arbovirus activity, rather than DENV specific dynamics. Nonetheless, we believe our methods and results provide useful insight into the patterns of mosquito-driven disease activity that can be used for surveillance and mitigation efforts.

The motivation for this work was to identify relevant spatio-temporal structure in historical DENV case data that could be leveraged for forecasting efforts. First, separating a time series into decomposed features has precedent in the field of forecasting. Brooks et al. established an empirical Bayes approach for influenza forecasting that used distributions of historical shape, noise level, peak height, peak week, and pacing transformations [62]. This technique was applied to predict DENV risk during the 2014 World Cup in Brazil [8]. Second, incorporating spatio-temporal structure into forecasting models has been shown to improve influenza predictions [63]. Our spatial clustering results can provide direction in how to incorporate current and historical spatial information in an informed and efficient way. Finally, our results also revealed that broad capturing and integration of disparate data streams are beneficial for explaining disease dynamics, and their utility in forecasting systems should continue to be explored [9, 10]. Here, we found that an individual time series data stream, such as temperature, can be used in numerous ways, whether as a standard summary statistic (e.g., mean), or as more complex dynamic measurements (e.g., intensity). Yet, the challenge remains that introducing more data streams and measurements increases the potential source of biases and risks obfuscating potential mechanistic insights that lend a model credibility. The increasing interface between infectious disease modelers, public health officials, and decision makers could guide the balance of incorporating big data and techniques while producing effective operational surveillance and forecasting systems. In addition to applying these results to mesoregion level forecasting efforts, future studies could consider how climate-change induced environmental changes may influence outbreak properties across the country.

## Supporting information

S1 Data Supporting files for analyses and figures. (ZIP)

S1 Fig Correlations between the mean values of the environmental variables. The diagonal shows the distribution of normalized values. The upper triangle contains the Pearson’s correlation value between two environmental variables.
(TIF)

S2 Fig Correlations between the intensity values of the environmental variables. The diagonal shows the distribution of normalized values. The upper triangle contains the Pearson’s correlation value between two environmental variables.
(TIF)

S3 Fig Correlations between the seasonality values of the environmental variables. The diagonal shows the distribution of normalized values. The upper triangle contains the Pearson’s correlation value between two environmental variables.
(TIF)

S4 Fig Correlations between pairwise correlations of environmental phase angles. The diagonal shows the distribution of normalized pairwise correlations of a specific environmental variable’s phase angle across all combinations of mesoregions. The upper triangle contains the Pearson’s correlation between two different environmental variables’ pairwise correlations.
(TIF)

S5 Fig Correlations between pairwise correlations of environmental trends. The diagonal shows the distribution of the normalized pairwise correlations of a specific environmental variable’s trends across all combinations of mesoregions. The upper triangle contains the Pearson’s correlation between two different environmental variables’ pairwise correlations.
(TIF)

S6 Fig Spatial variation in the mean values of the environmental variables. All variables have been normalized such that darker areas represent higher values and lighter areas represent lower values. The underlying shapefiles with political boundaries are publicly and freely available at Instituto Brasileiro de Geografia e Estatística (IBGE) http://downloads.ibge.gov.br/downloads_geociencias.htm.
(TIF)

S7 Fig Spatial variation in the intensity values of the environmental variables. All variables have been normalized such that darker areas represent higher values and lighter areas represent lower values. The underlying shapefiles with political boundaries are publicly and freely available at Instituto Brasileiro de Geografia e Estatística (IBGE) http://downloads.ibge.gov.br/downloads_geociencias.htm.
(TIF)

S8 Fig Spatial variation in the seasonality values of the environmental variables. All variables have been normalized such that darker areas represent higher values and lighter areas represent lower values. The underlying shapefiles with political boundaries are publicly and freely available at Instituto Brasileiro de Geografia e Estatística (IBGE) http://downloads.ibge.gov.br/downloads_geociencias.htm.
(TIF)

S9 Fig Spatial variation in the human-risk factors. All variables have been normalized such that darker areas represent higher values and lighter areas represent lower values. The underlying shapefiles with political boundaries are publicly and freely available at Instituto Brasileiro de Geografia e Estatística (IBGE) http://downloads.ibge.gov.br/downloads_geociencias.htm.
(TIF)

S10 Fig In-sample and out-of-sample fits from the best-fit model. Each panel shows one of the 100 LASSO fits. The orange dots represent the in-sample target values versus the fitted values; the grey dots represent the out-of-sample target values versus the fitted values. The smooth lines show the relationship of yx̃, while the dotted line indicates a perfect agreement between the target and fitted values.
(TIF)

S11 Fig Sensitivity of the outbreak intensity threshold. (A) Mean intensity values across all mesoregions when excluding annual outbreaks in mesoregions that do not exceed an annual threshold of 5–200 total DENV cases. By including years when a mesoregion has fewer than 10 cumulative DENV cases, the resulting mean intensity distribution is highly skewed. Using thresholds of 50–200 cases results in similar mean intensity distributions. A threshold of 50 completely excludes five mesoregions, a threshold of 100 completely excludes seven mesoregions, and thresholds of 150 and 200 completely exclude eight mesoregions. (B) For the remaining 129 mesoregions, when using a threshold of 150 annual DENV cases, the mean intensity values computed when excluding years with fewer than 150 cases are higher than when including all outbreaks. Orange dots represent mesoregions that had at least one year with fewer than 150 DENV cases. Purple dots represent mesoregions where there were at least 150 DENV cases in all years across the six-year study period. (C) Including years when a mesoregion had fewer than 150 total DENV cases results in a skewed distribution of the overall mean intensity values. In the main manuscript, we use the mean intensity distribution corresponding to the blue line, excluding outbreak years in which a mesoregion had fewer than 150 DENV cases.
(TIF)
