# Supplementary figures and images for: Using heterogeneous data to identify signatures of dengue outbreaks at fine spatio-temporal scales across Brazil
Source: PLoS Negl Trop Dis. 2021 May 21;15(5):e0009392. doi: 10.1371/journal.pntd.0009392 (PMC8174735; doi:10.1371/journal.pntd.0009392)

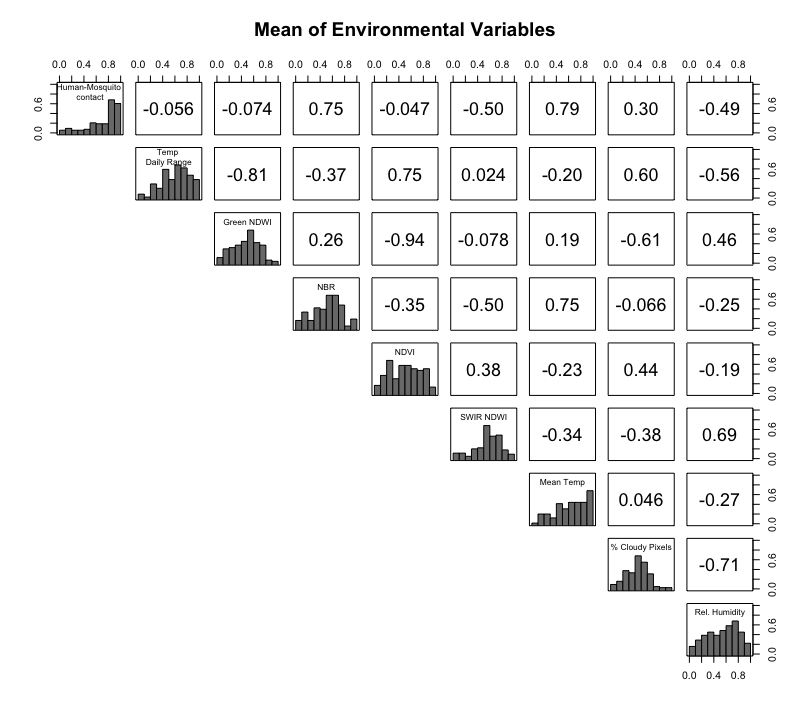

Supplement: S1 Fig — The diagonal shows the distribution of normalized values. The upper triangle contains the Pearson’s correlation value between two environmental variables. (TIF) [file pntd.0009392.s002.tif]

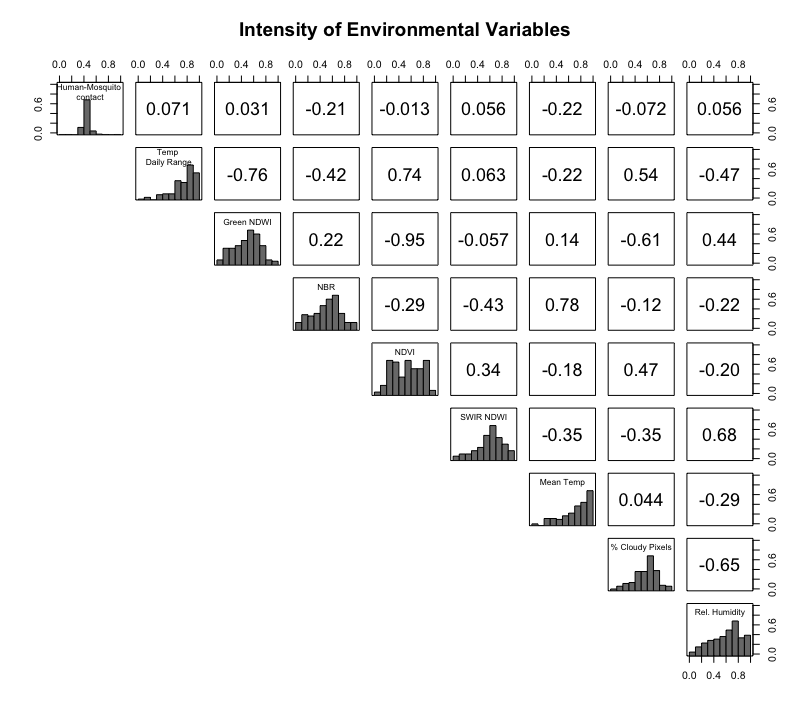

Supplement: S2 Fig — The diagonal shows the distribution of normalized values. The upper triangle contains the Pearson’s correlation value between two environmental variables. (TIF) [file pntd.0009392.s003.tif]

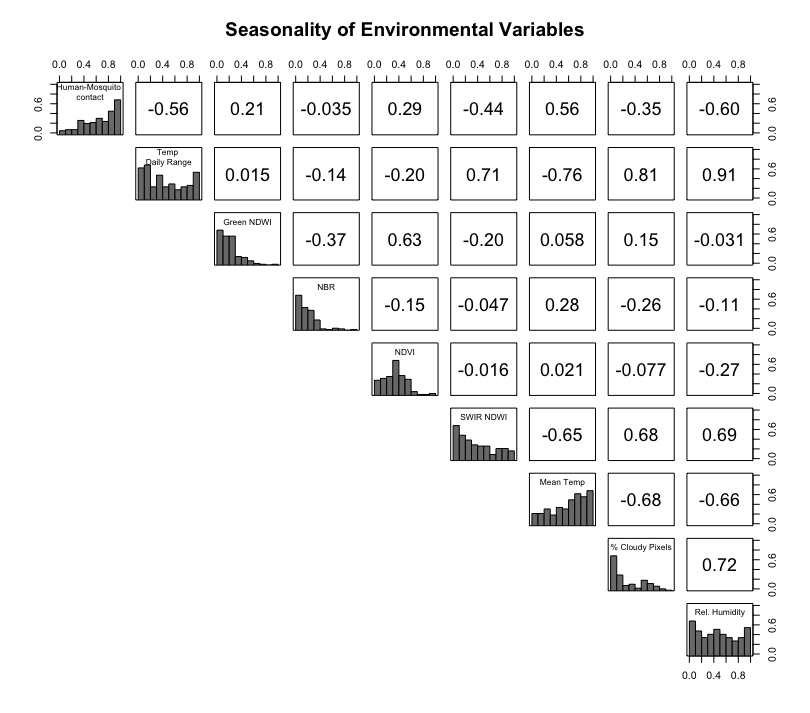

Supplement: S3 Fig — The diagonal shows the distribution of normalized values. The upper triangle contains the Pearson’s correlation value between two environmental variables. (TIF) [file pntd.0009392.s004.tif]

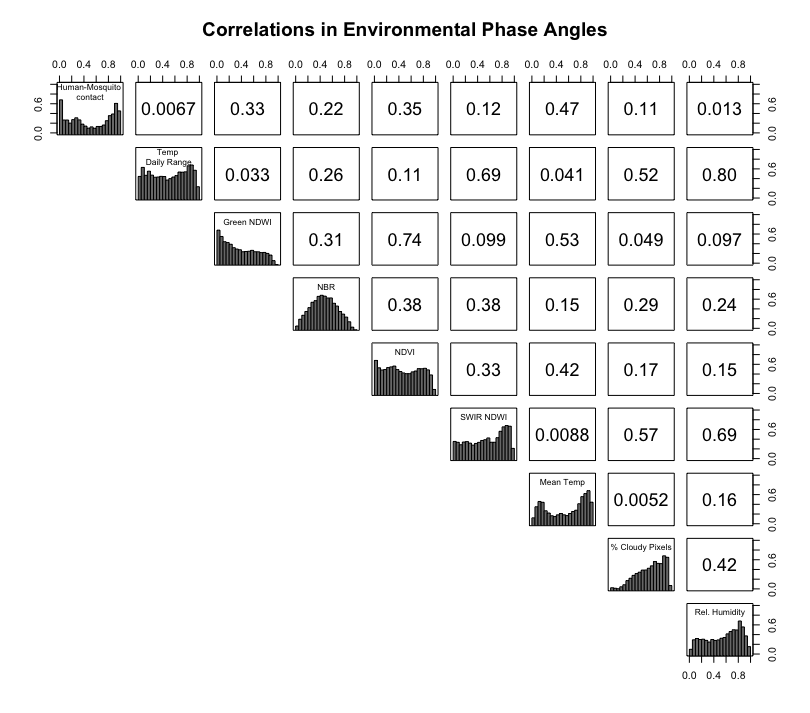

Supplement: S4 Fig — The diagonal shows the distribution of normalized pairwise correlations of a specific environmental variable’s phase angle across all combinations of mesoregions. The upper triangle contains the Pearson’s correlation between two different environmental variables’ pairwise correlations. (TIF) [file pntd.0009392.s005.tif]

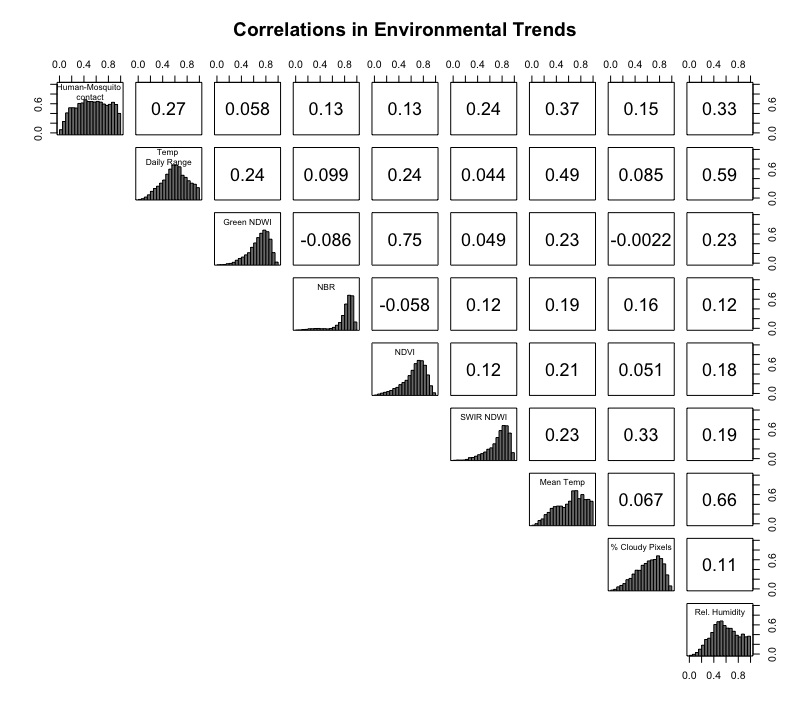

Supplement: S5 Fig — The diagonal shows the distribution of the normalized pairwise correlations of a specific environmental variable’s trends across all combinations of mesoregions. The upper triangle contains the Pearson’s correlation between two different environmental variables’ pairwise correlations. (TIF) [file pntd.0009392.s006.tif]

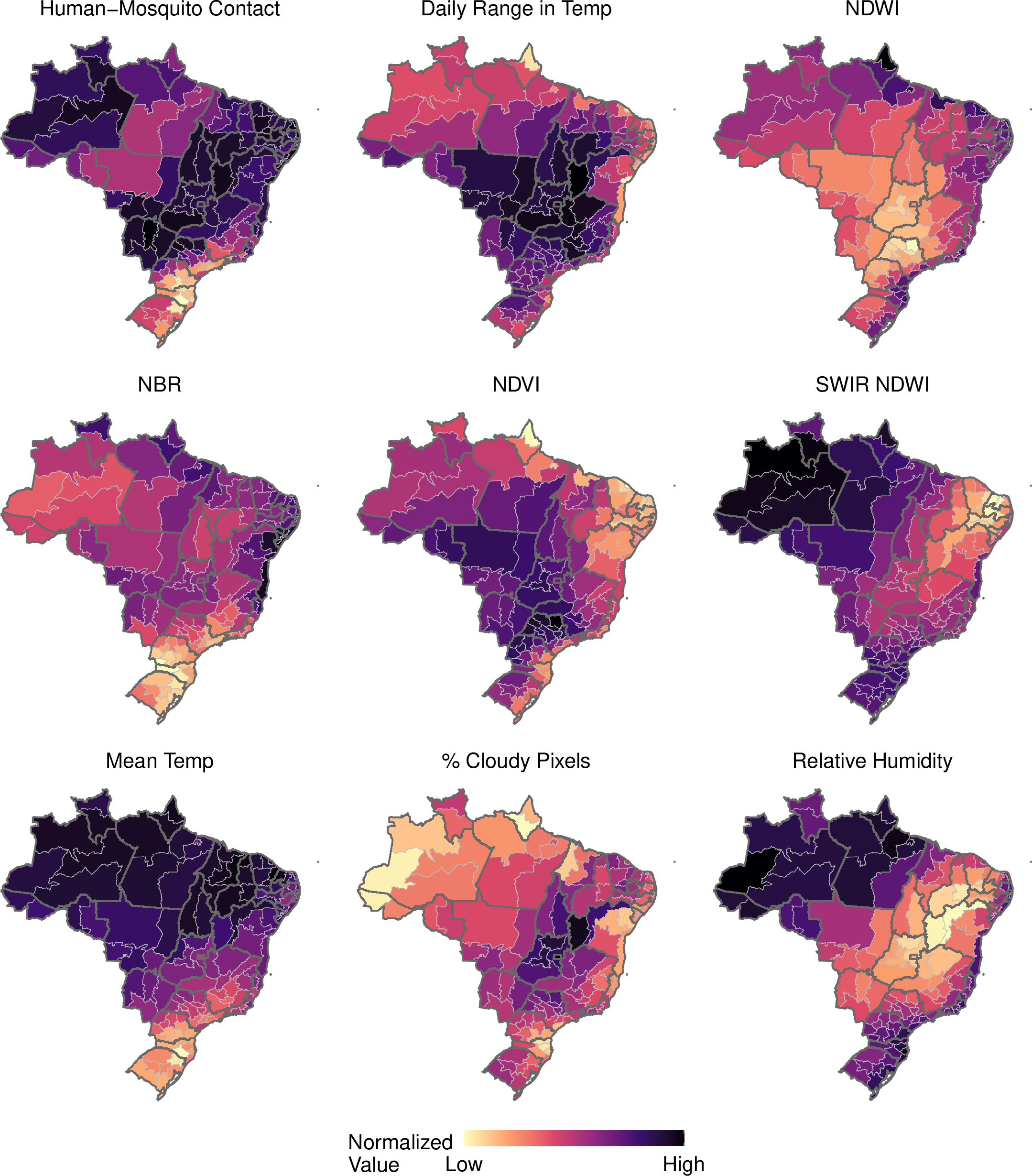

Supplement: S6 Fig — All variables have been normalized such that darker areas represent higher values and lighter areas represent lower values. The underlying shapefiles with political boundaries are publicly and freely available at Instituto Brasileiro de Geografia e Estatística (IBGE) http://downloads.ibge.gov.br/downloads_geociencias.htm. (TIF) [file pntd.0009392.s007.tif]

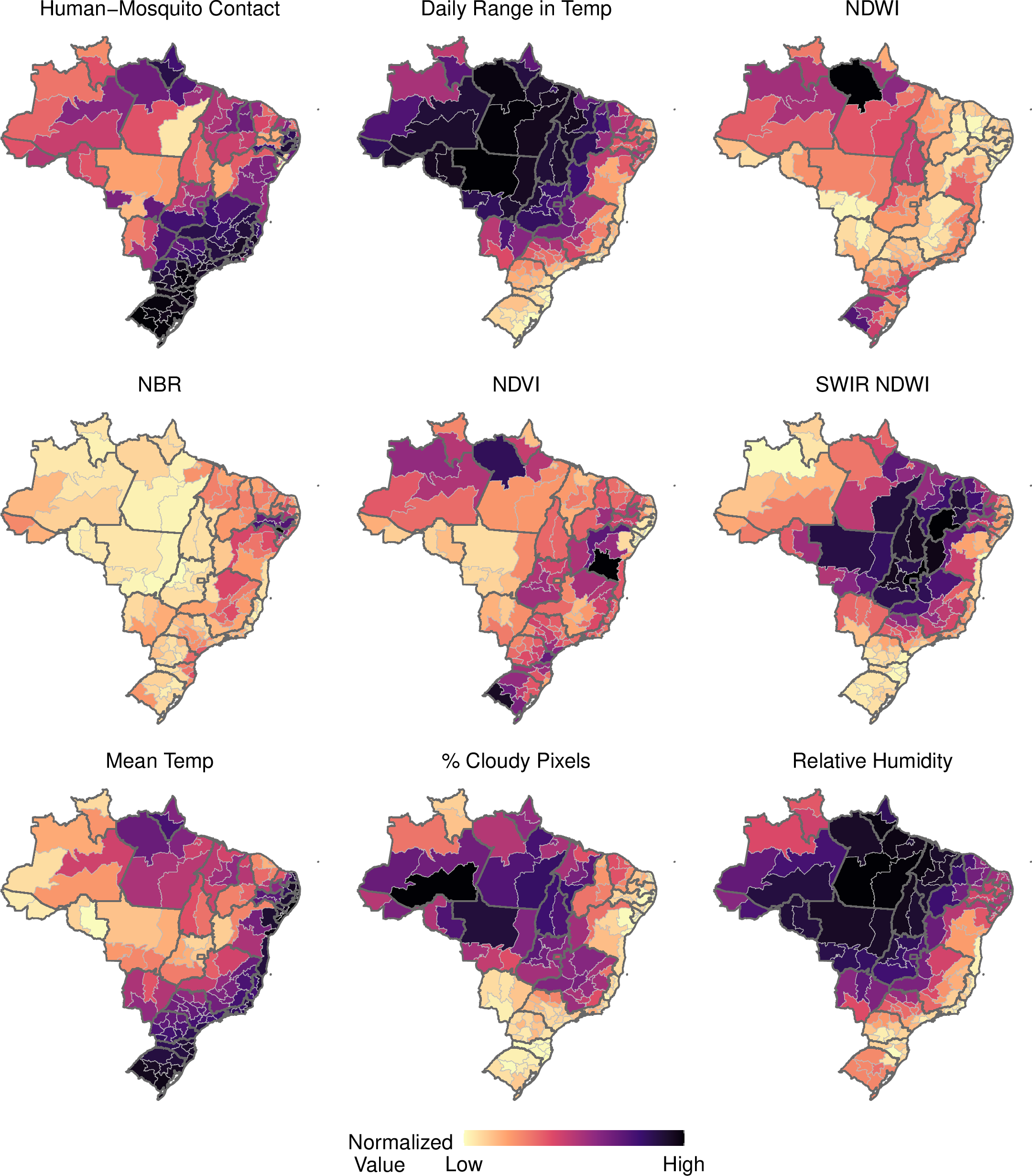

Supplement: S7 Fig — All variables have been normalized such that darker areas represent higher values and lighter areas represent lower values. The underlying shapefiles with political boundaries are publicly and freely available at Instituto Brasileiro de Geografia e Estatística (IBGE) http://downloads.ibge.gov.br/downloads_geociencias.htm. (TIF) [file pntd.0009392.s008.tif]

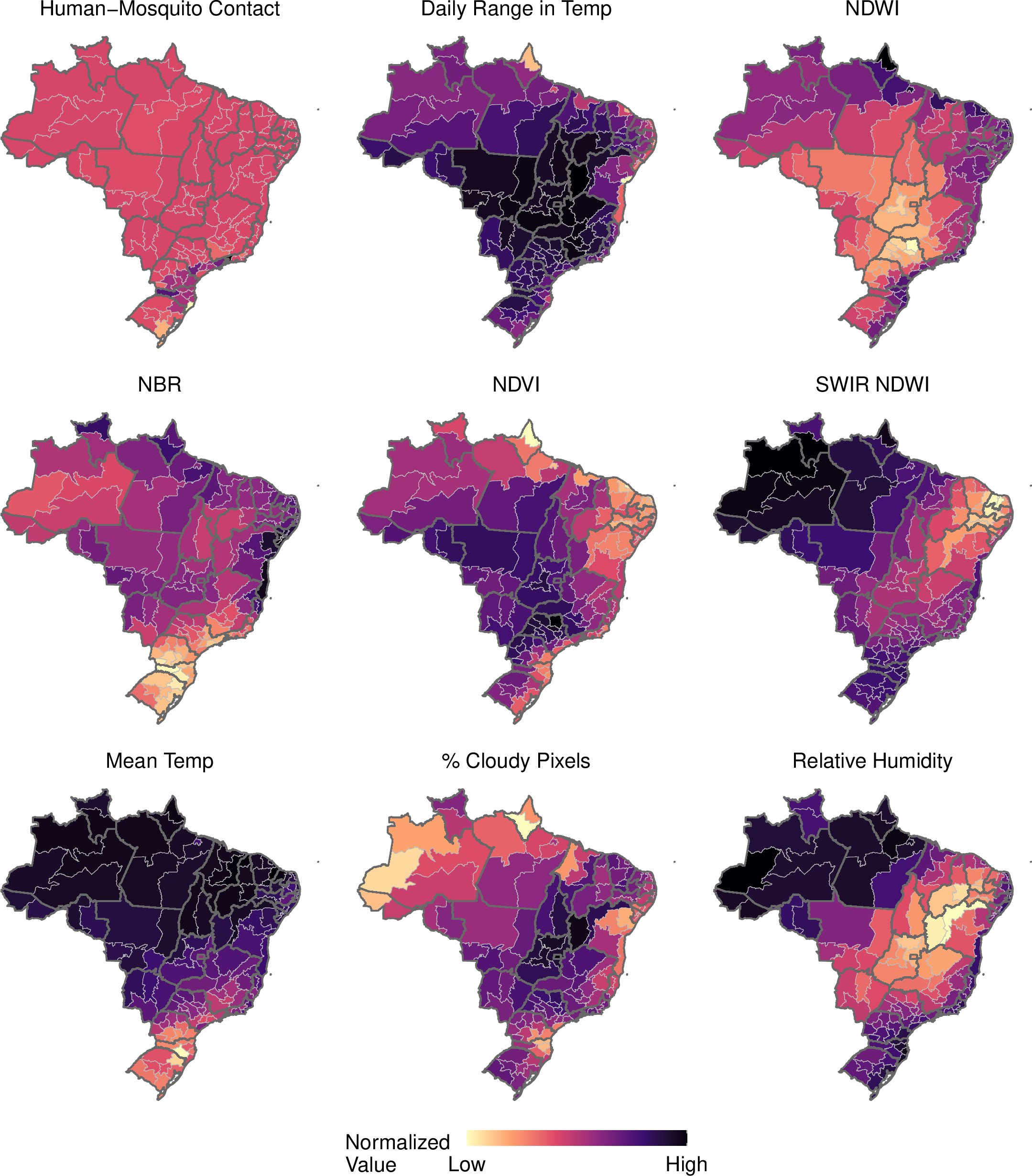

Supplement: S8 Fig — All variables have been normalized such that darker areas represent higher values and lighter areas represent lower values. The underlying shapefiles with political boundaries are publicly and freely available at Instituto Brasileiro de Geografia e Estatística (IBGE) http://downloads.ibge.gov.br/downloads_geociencias.htm. (TIF) [file pntd.0009392.s009.tif]

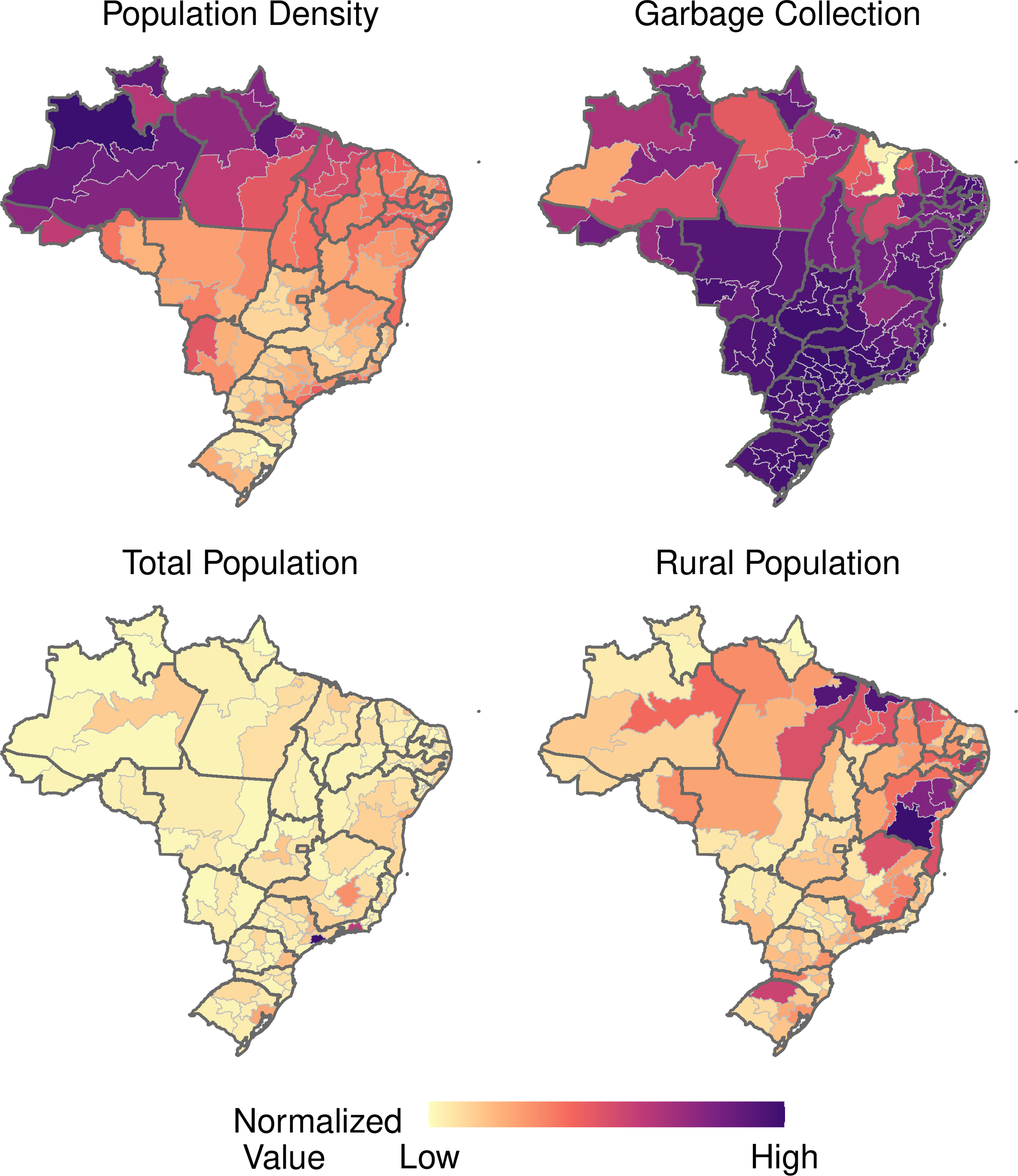

Supplement: S9 Fig — All variables have been normalized such that darker areas represent higher values and lighter areas represent lower values. The underlying shapefiles with political boundaries are publicly and freely available at Instituto Brasileiro de Geografia e Estatística (IBGE) http://downloads.ibge.gov.br/downloads_geociencias.htm. (TIF) [file pntd.0009392.s010.tif]

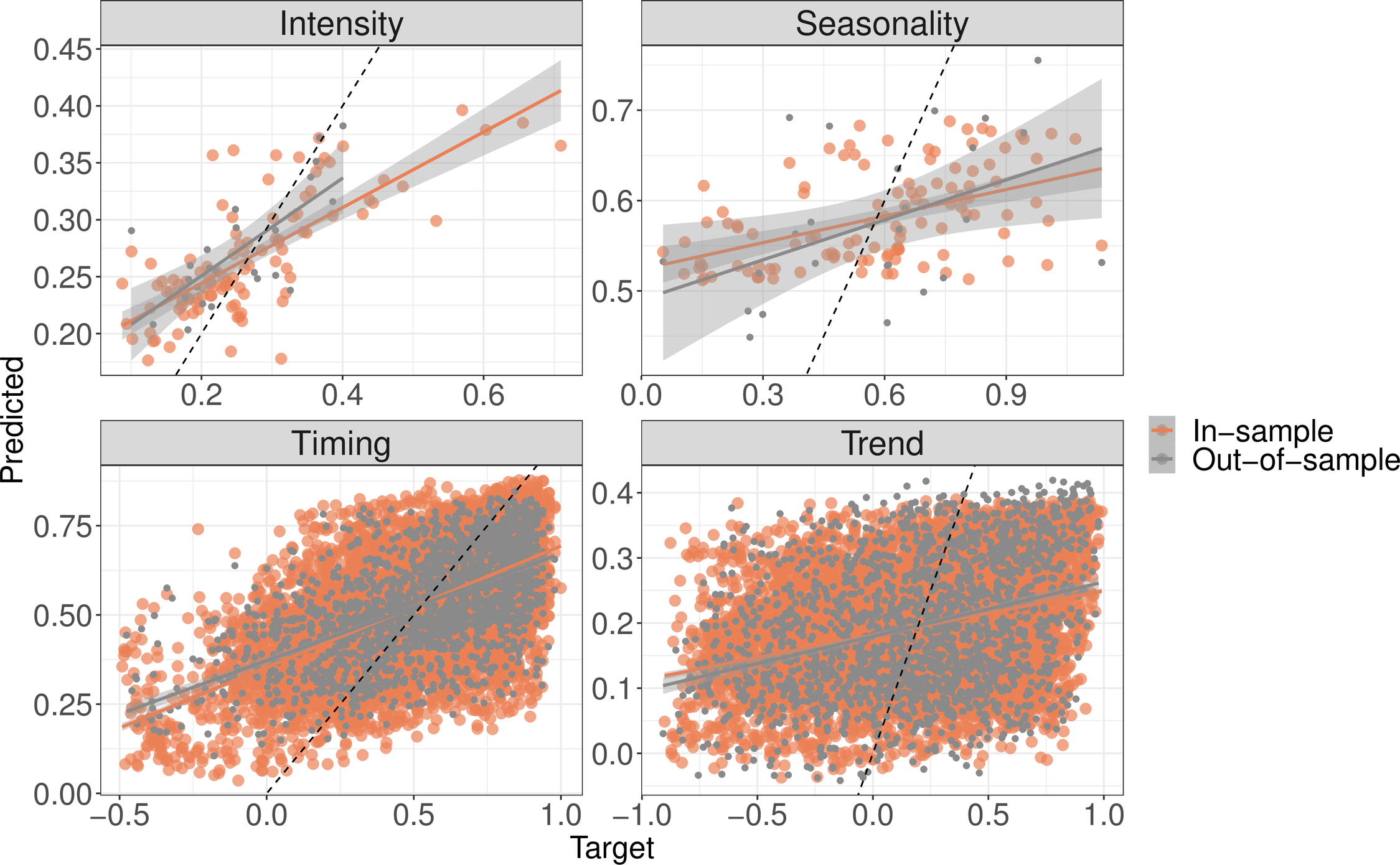

Supplement: S10 Fig — Each panel shows one of the 100 LASSO fits. The orange dots represent the in-sample target values versus the fitted values; the grey dots represent the out-of-sample target values versus the fitted values. The smooth lines show the relationship of yx̃, while the dotted line indicates a perfect agreement between the target and fitted values. (TIF) [file pntd.0009392.s011.tif]

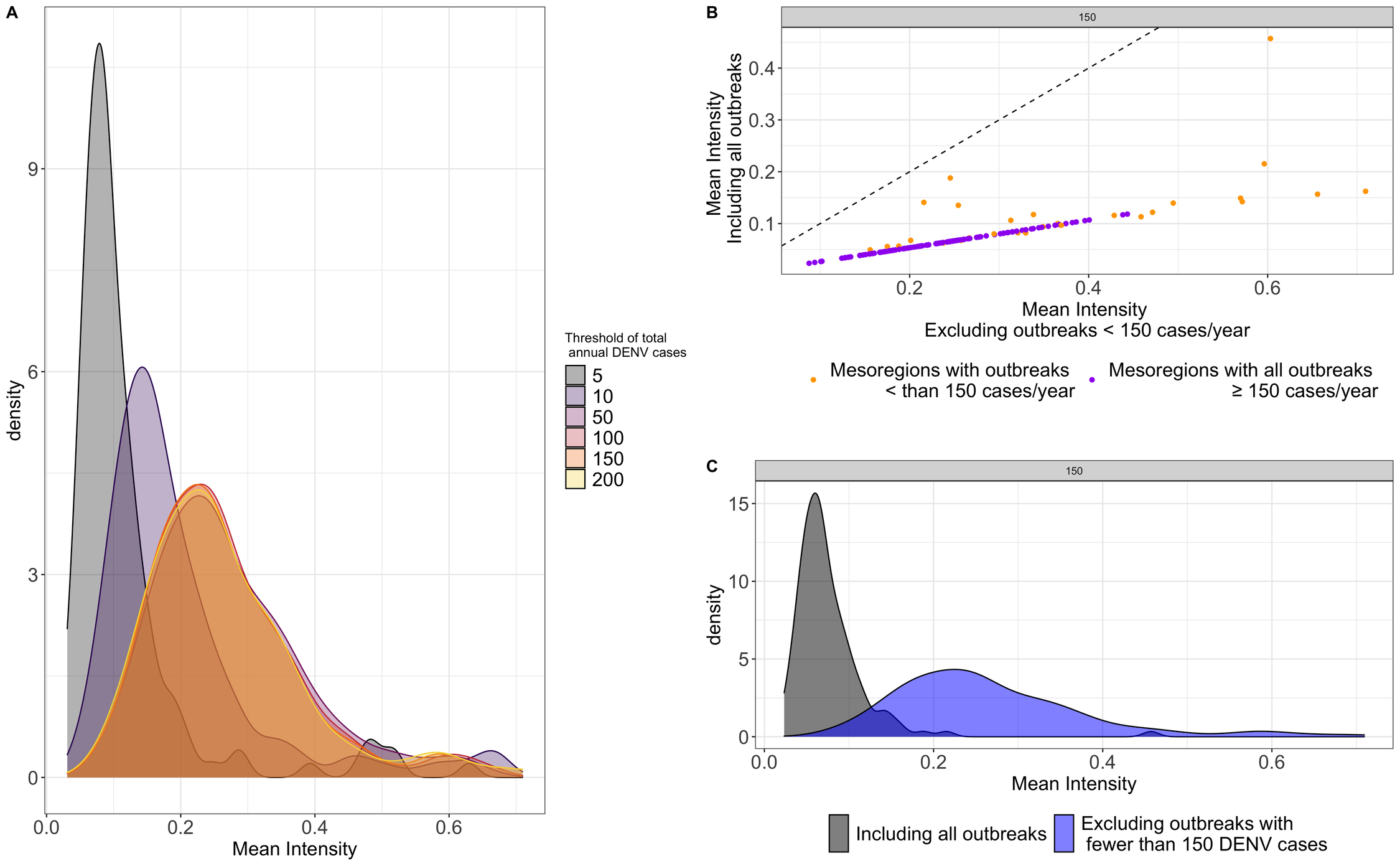

Supplement: S11 Fig — (A) Mean intensity values across all mesoregions when excluding annual outbreaks in mesoregions that do not exceed an annual threshold of 5–200 total DENV cases. By including years when a mesoregion has fewer than 10 cumulative DENV cases, the resulting mean intensity distribution is highly skewed. Using thresholds of 50–200 cases results in similar mean intensity distributions. A threshold of 50 completely excludes five mesoregions, a threshold of 100 completely excludes seven mesoregions, and thresholds of 150 and 200 completely exclude eight mesoregions. (B) For the remaining 129 mesoregions, when using a threshold of 150 annual DENV cases, the mean intensity values computed when excluding years with fewer than 150 cases are higher than when including all outbreaks. Orange dots represent mesoregions that had at least one year with fewer than 150 DENV cases. Purple dots represent mesoregions where there were at least 150 DENV cases in all years across the six-year study period. (C) Including years when a mesoregion had fewer than 150 total DENV cases results in a skewed distribution of the overall mean intensity values. In the main manuscript, we use the mean intensity distribution corresponding to the blue line, excluding outbreak years in which a mesoregion had fewer than 150 DENV cases. (TIF) [file pntd.0009392.s012.tif]
